# Progress Toward Epigenetic Targeted Therapies for Childhood Cancer

**DOI:** 10.3390/cancers16244149

**Published:** 2024-12-12

**Authors:** Athanasia Liapodimitri, Ashley R. Tetens, Jordyn Craig-Schwartz, Kayleigh Lunsford, Kegan O. Skalitzky, Michael A. Koldobskiy

**Affiliations:** 1 Division of Pediatric Oncology, Department of Oncology, School of Medicine, Johns Hopkins University, Baltimore, MD 21287, USA; aliapod1@jh.edu (A.L.); atetens1@jhu.edu (A.R.T.); jcraigs1@jhu.edu (J.C.-S.); klunsfo1@jhmi.edu (K.L.); kskalit1@jhmi.edu (K.O.S.); 2 Sidney Kimmel Comprehensive Cancer Center, School of Medicine, Johns Hopkins University, Baltimore, MD 21287, USA

**Keywords:** epigenetics, pediatric cancer, DNA methylation, histone modifications, epigenetic therapy

## Abstract

Among the most significant discoveries from cancer genomics efforts has been the critical role of epigenetic dysregulation in cancer development and progression. Studies across diverse cancer types have revealed frequent mutations in genes encoding epigenetic regulators, alterations in DNA methylation and histone modifications, and a dramatic reorganization of chromatin structure. Epigenetic changes are especially relevant to pediatric cancers, which are often characterized by a low rate of genetic mutations. The inherent reversibility of epigenetic lesions has led to an intense interest in the development of epigenetic targeted therapies. Additionally, the recent appreciation of the interplay between the epigenome and immune regulation has sparked interest in combination therapies and synergistic immunotherapy approaches. Further, the recent appreciation of epigenetic variability as a driving force in cancer evolution has suggested new roles for epigenetic therapies in limiting plasticity and resistance. Here, we review recent progress and emerging directions in the development of epigenetic targeted therapeutics and their promise across the landscape of childhood cancers.

## Simple Summary

Drugs targeting regulators of the epigenome hold great promise in cancer and have been approved for specific indications in adult oncology. Epigenetic changes are especially critical to pediatric cancers, which often have a low rate of genetic mutation. Here we discuss potential targets for epigenetic therapies in childhood cancer and summarize progress toward the clinical translation of preclinical advances in the field.

## 1. Introduction

Epigenetics refers to modifications to the genetic material that influence gene activity and that are heritable during cell division, but do not alter the DNA sequence itself. These include reversible chemical modifications of the DNA and histones that alter the chromatin state and accessibility to DNA binding proteins, such as transcription factors. The epigenetic code is regulated by a dynamic network of enzymes, including those that ‘write’ or deposit epigenetic chemical marks, those that ‘read’ or recognize these modifications, and those that ‘erase’ or remove the marks. Epigenetic modifiers are critical regulators of cell fate determination, maintenance of cellular identity, spatiotemporal regulation of gene expression, and transcriptional responses to stimuli and environmental inputs. Since the initial discovery of altered DNA methylation in cancer by Feinberg and Vogelstein in 1983 [1], the field of cancer epigenetics has grown rapidly, and it has been established that epigenetic dysregulation is a fundamental hallmark of cancer [2,3,4]. Genomic studies across a variety of cancer types have identified frequent mutations in genes encoding epigenetic regulators [5], widespread alterations in DNA methylation and histone modifications [6], and a dramatic reorganization of chromatin structure [7].

Epigenetic alterations are especially relevant to pediatric cancers, which are often characterized by a very low rate of genetic mutation [8,9]. Indeed, some aggressive childhood cancer types have remarkably simple genomes, with their few genetic hits confined to regulators of the epigenome. Prominent examples include malignant rhabdoid tumors, driven by biallelic loss of the chromatin remodeler *SMARCB1*, with few other recurrent genetic mutations [8,10], diffuse midline glioma (DMG), driven by oncogenic histone mutations, leading to altered histone methylation [11,12], and pediatric hindbrain ependymomas, which have a minimal mutational burden and, instead, are driven by altered DNA methylation [9]. The central role of epigenetic derangements across childhood cancers, combined with the inherent reversibility of epigenetic lesions, has sparked an intense interest in therapies targeted at the epigenome. To date, however, the approved epigenetic agents are largely limited to narrow adult oncology indications. In this review, we evaluate recent progress in the preclinical and clinical development of epigenetic therapies for childhood cancer. We consider emerging concepts related to the epigenetic regulation of phenotypic variability and plasticity, and the interplay of the epigenome and immune regulation, to propose future directions and combination therapies that harness the promise of epigenetic therapeutics for childhood cancer.

## 2. Targeting the Epigenetic Machinery

**Figure 1:**
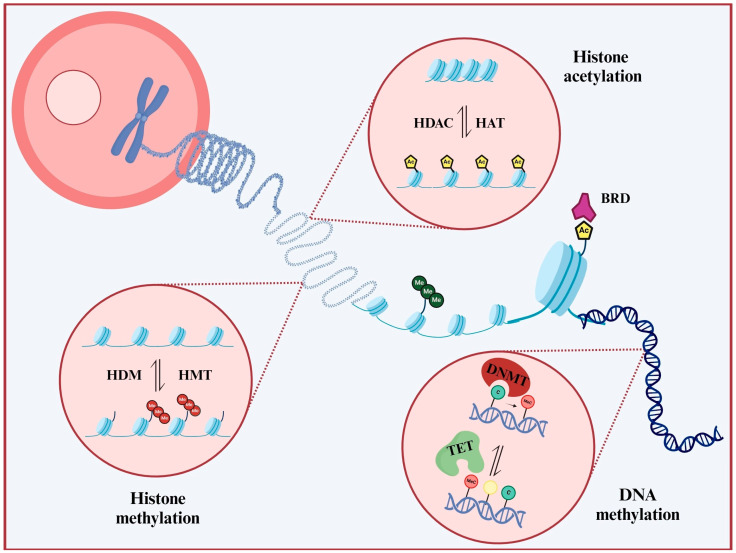
Unwinding epigenetic control of chromatin. Prominent epigenetic alterations include DNA methylation and histone modifications, including histone methylation and acetylation. Specific enzymes, such as DNA Methyltransferases (DNMTs), Histone Methyltransferases (HMTs), and Histone Acetylases (HATs), ‘write’ these modifications, while enzymes such as Ten-Eleven Translocation protein methylcytosine dioxygenases (TETs), Histone Demethylases (HDMs), and Histone Deacetylases (HDACs) are responsible for ‘erasing’ them. Complementary modules, such as Bromodomains (BRDs), act as ‘readers’, recognizing and binding to modified residues.

Epigenetic regulation occurs at multiple levels, involving hundreds of enzymes and regulatory complexes. Key mechanisms include direct DNA modification, histone alterations, chromatin remodeling, and changes in the organization of DNA within chromatin structures (Figure 1).

**Figure 2:**
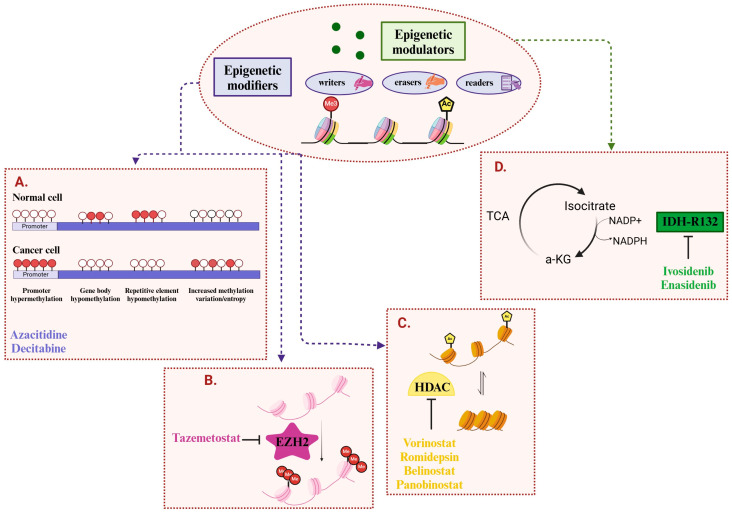
Common epigenetic alterations in cancer and associated FDA-approved drugs. Epigenetic modifiers include writers, erasers, and readers of epigenetic marks, and directly alter the epigenome. Epigenetic modulators function upstream of epigenetic modifiers, and integrate environmental and metabolic inputs toward the epigenome: (**A**) DNA methylation in cancer vs. normal cells (methylated sites are represented by the red circles; unmethylated sites are represented by the white circles). Hallmark alterations in cancer are shown, including promoter hypermethylation, gene body and repetitive element hypomethylation, and increased methylation entropy. DNA methylation in cancer can be targeted by DNMT inhibitors, such as the FDA-approved azacitidine and decitabine. (**B**) EZH2, the catalytic subunit of the PRC2 complex, can be mutated or overexpressed in various cancer types, and it facilitates the tri-methylation of histone H3 lysine 27, serving as a target for EZH2 inhibitors, such as tazemetostat. (**C**) HDACs remove acetyl groups from histone lysine residues, typically favoring an inactive chromatin conformation. HDACs are targeted by several FDA-approved inhibitors, such as vorinostat, romidepsin, belinostat, and panobinostat. (**D**) Mutant IDH1/2 is a prominent example of an altered epigenetic modulator, producing an oncometabolite that inhibits epigenetic modifiers, such as TET enzymes and histone lysine demethylases. Mutant IDH inhibitors include ivosidenib and enasidenib. TCA, tricarboxylic acid cycle; a-KG, alpha-ketoglutarate.

Genes that shape the cancer epigenome can be organized within an epigenetic classification system. This includes epigenetic modifiers—commonly mutated in cancer—such as regulators of DNA methylation and histone modification, alongside epigenetic modulators, which have roles in signaling or metabolic pathways but exert an indirect influence on the epigenome [3]. Below, we briefly review relevant mechanisms underlying epigenetic regulation that are targeted by current epigenetic therapies (Figure 2).

### 2.1. DNA Methylation

DNA methylation is a critically important modification in transcriptional regulation, consisting of methylation at the 5-carbon of cytosine residues, predominantly within CpG dinucleotides. Methylation of CpG islands in gene promoter regions is classically linked to transcriptional repression, but diverse functions of DNA methylation in the regulation of differentiation and development are now appreciated [13,14].

Altered DNA methylation is a universal feature of cancer, and, indeed, it was the initial discovery that launched the field of cancer epigenetics [1]. Global DNA hypomethylation has commonly been observed in cancer cells, correlating with the overexpression of oncogenic drivers [1,15,16], while hypermethylation in promoters of tumor suppressor genes has been linked to crucial gene silencing events of targets such as *Rb* [17] and *p16* [18]. In addition to changes in mean methylation, recent work has highlighted that cancer cells exhibit markedly increased variability of DNA methylation patterns, suggesting a role for stochastic variation in generating epigenetic diversity [19]. Thus, it is not surprising that changes in DNA methylation are thought to be among the earliest changes in tumor initiation [20].

From a mechanistic perspective, DNA Methyltransferases DNMT3A and DNMT3B are responsible for de novo deposition of methylation marks on unmethylated CpGs, while DNMT1 primarily operates during replication, methylating hemi-methylated DNA, thus participating in the inheritance of existing methylation marks [21,22]. Interestingly, DNMT3A has been shown to be crucial in Hematopoietic Stem Cell (HSC) differentiation, and loss of DNMT3A drives the expansion of the stem cell pool [23]. DNMT3A inactivation confers a preleukemic phenotype on murine HSCs, predisposing mice to developing a wide range of malignancies, of both myeloid and lymphoid origin [24]. Supporting these findings, DNMT3A mutations are frequently observed in adult patients with de novo Acute Myeloid Leukemia (AML) [25,26,27,28], but they are rarely seen in pediatric AML [29].

Enzymes involved in ‘erasing’ DNA methylation marks include the Ten-Eleven Translocation (TET) enzymes (TET1, TET2, and TET3) [30], which catalyze stepwise oxidation of 5-methylcytosine (5mC) to 5-hydroxymethylcytosine (5hmC) in an α-ketoglutarate (α-KG)-dependent manner [31]. The necessary α-KG is generated by isocitrate dehydrogenase (IDH) enzymes within the tricarboxylic acid cycle (TCA) cycle. Importantly, tumor-associated hotspot mutations in IDH1/2 can drive the conversion of α-KG into the oncometabolite D-2-hydroxyglutarate (2-HG), inhibiting TET function and resulting in hypermethylation [32].

Alterations involving the DNA-demethylating machinery have been linked to multiple cancers. TET1 has been identified as the translocation partner gene of the Mixed Lineage Leukemia gene (MLL) in a subset of AML and B-cell Acute Lymphoblastic Leukemia (ALL) patients [33,34,35]. TET1 is a direct target of MLL fusion proteins and is significantly upregulated in MLL-rearranged leukemias [36]. Conversely, TET1 loss promotes B cell malignancies [37], while acute germline deletion of TET activity leads to rapid development of aggressive myeloid leukemia in mice [38], suggesting a potential tumor suppressor role. TET2 mutations have been linked to a wide spectrum of myeloid cancers [39], and correlate with decreased overall survival in AML [40].

IDH1 mutations resulting in the overproduction of 2-HG contribute to initiation and progression of myeloid malignancies and gliomas [41]. IDH1 mutations drive a glioma hypermethylator phenotype [42,43,44], and now define distinct subtypes of pediatric and adult glioma [45]. IDH1/2 oncogenic activity in leukemia has also been validated in vivo, with IDH mutation being identified as an early event in leukemogenesis, impeding hematopoietic differentiation and leading to myeloid skewing [46,47].

Given the reversible nature of DNA methylation and its prominent dysregulation in cancer, multiple therapeutics aimed at the DNA methylation machinery have been developed. Currently, two hypomethylating agents, 5-azacitidine and 5-aza-2′-deoxycytidine (decitabine), are approved for treating myelodysplastic syndrome, adult AML [48], and chronic myelomonocytic leukemia (CMML) [49,50].

In childhood cancer, hypomethylating agents have primarily been studied in combination with other agents. A phase I trial by the Therapeutic Advances in Childhood Leukemia and Lymphoma (TACL) consortium investigated the combination of decitabine with the HDAC inhibitor vorinostat, followed by conventional chemotherapy (FLAG regimen), in children with relapsed/refractory AML (NCT02412475). This trial showed a CR/CRi rate of 54%, with 90% of the responders achieving Minimal Residual Disease (MRD) negativity (<0.1% by flow cytometry) despite the high disease burden. Greater responses were observed in patients with documented epigenetic alterations [51]. A follow-up study, involving pediatric patients with relapsed/refractory or therapy-related myeloid malignancies not included in the TACL study, also yielded favorable results, particularly in patients with epigenetic alterations, therapy-related AML, and refractory disease (NCT01483690) [52]. Additionally, a phase I multicenter trial using decitabine as a single agent prior to standard chemotherapy in children with newly diagnosed AML demonstrated favorable safety and tolerability and demonstrated changes in DNA methylation patterns (NCT01177540) [53].

Similarly, in a phase II trial for pediatric relapsed/refractory ALL, the combination of decitabine and vorinostat, followed by four-drug re-induction chemotherapy, including doxorubicin, demonstrated clinical benefit and tolerability (NCT00882206) [54]. Notably, methylation differences following decitabine treatment distinguished responders from non-responders, indicating a potential link between methylation patterns and clinical benefit, mapping to genes within biologically significant networks. However, a subsequent study incorporating decitabine/vorinostat pre-treatment and four-drug re-induction including mitoxantrone reported an exceedingly high rate of infectious complications, ultimately determining that this approach was not feasible, despite favorable response rates and pharmacodynamics (NCT01483690) [55].

Studies of hypomethylating agents for pediatric solid tumors are more limited. A Children’s Oncology Group phase I clinical trial evaluated decitabine followed by doxorubicin and cyclophosphamide for children with refractory neuroblastoma or other solid tumors (NCT00075634) [56]. Low-dose decitabine in combination with chemotherapy had tolerable toxicity, but higher-dose decitabine had dose-limiting hematologic toxicities. On-target effects were demonstrated, with sustained demethylation of the tumor antigen MAGE-1 and changes in gene expression [56]. A retrospective analysis of patients with refractory rhabdoid tumors, who were administered decitabine with chemotherapy upon relapse or progression, reported radiographic responses in a subset of patients, with prolonged time to progression and overall survival in responders, and a relationship between response and tumor methylation signature [57].

In vitro studies underscore the immunomodulatory properties of hypomethylating drugs, such as enhancing neoantigen expression in glioblastoma [58] and upregulating immune signaling in DMG [59,60]. This has led to interest in combinations of decitabine with immune checkpoint blockade, such as programmed cell death 1 (PD-1) antagonists [61].

In the case of IDH1/2 mutation in both AML and brain tumors, several novel therapies have been developed. Vorasidenib, a brain-penetrant dual inhibitor of mutant IDH1 and IDH2 enzymes, enhanced progression-free survival of patients with residual or recurrent grade 2 IDH-mutant glioma (NCT04164901) [62,63]. Similarly, ivosidenib, an oral mutant IDH1 inhibitor, has shown durable remissions and favorable tolerability in relapsed or refractory IDH1-mutated AML (NCT02074839) [64]. Enasidenib, a selective IDH2 inhibitor, also demonstrated promising remission outcomes, driven by differentiation rather than cytotoxicity in patients with IDH2-mutant relapsed or refractory AML (NCT01915498) [65], and proved effective as salvage therapy in heavily treated AML patients (NCT01915498, NCT02577406) [66,67]. Furthermore, phase I trials of ivosidenib and enasidenib combined with chemotherapy in newly diagnosed AML patients have yielded favorable clinical responses (NCT02632708) [68]. Both drugs have also been found to be effective in combination therapies of ivosidenib with azacitidine and venetoclax for IDH1-mutated AML (NCT03471260, NCT02677922, NCT03683433) [69,70,71].

Overall, the altered DNA methylation landscape has a central role in cancer, and targeting the epigenetic modifiers (e.g., DNMTs) and modulators (e.g., IDH1/2) involved in DNA methylation regulation can provide an avenue to modify cancer-associated transcriptional changes. However, since DNA methylation constitutes only one layer of epigenetic regulation, its role in gene regulation must also be considered in the context of other epigenetic modifications [72].

### 2.2. Histone Modifications

#### 2.2.1. Histone Methylation—Polycomb Repressive Complex 2

Combinatorial modification of specific residues on histones creates a regulatory code that regulates gene expression in normal development and cancer [73]. A central player in this process is the Polycomb Repressive Complex 2 (PRC2), a chromatin-modifying complex responsible for gene silencing [3]. Many pathways associated with pediatric cancer converge on the disruption of PRC2 regulation, underscoring the instrumental role of PRC2 in shaping the epigenetic control between normal development and malignant growth [74,75].

**Figure 3:**
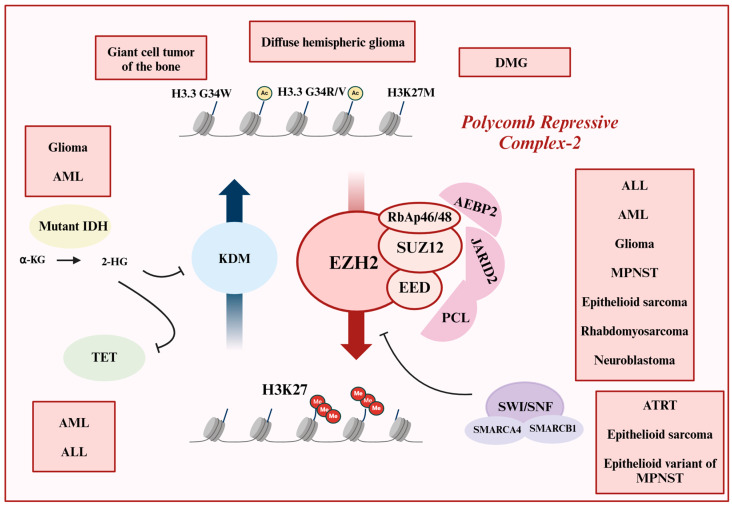
Convergence of epigenetic disruption in childhood cancer on PRC2. The PRC2 complex, with its catalytic subunit, EZH2, is responsible for the tri-methylation of lysine 27 on histone H3. Various mutations, including gain-of-function, loss-of-function, and overexpression, have been associated with numerous childhood cancers. ALL, AML, and glioma are examples of cancers linked to disrupted PRC2 function. The SWI/SNF complex, which typically antagonizes the PRC2 complex, is dysregulated in pediatric malignant rhabdoid tumors, such as Atypical Teratoid Rhabdoid Tumor (ATRT), as well as epithelioid sarcoma and the epithelioid variant of Malignant Peripheral Nerve Sheath Tumor (MPNST). Mutant IDH, found in glioma and AML, produces 2-HG, an oncometabolite that inhibits both KDM and TET enzymes. Additionally, oncohistone mutations exist in a variety of pediatric cancers, including giant cell tumors of the bone, diffuse hemispheric glioma, and DMG. In pediatric DMG, the H3K27M oncohistone antagonizes PRC2 activity. In summary, PRC2 serves as a central node in epigenetic dysregulation across diverse pediatric malignancies.

The PRC2 complex consists of four primary constitutive domains: Enhancer of Zeste Homolog 2 (EZH2), Embryonic Ectoderm Development (EED), Suppressor of Zeste 12 (SUZ12), and Retinoblastoma Suppressor Associated Protein 46/48 (RbAp46/48), supported by partners like AE (adipocyte enhancer)-binding protein 2 (AEBP2), Jumonji/AT-rich Interactive Domain 2 (JARID2), and polycomb-like (PCL) proteins 1, 2, and 3. EZH2, the core catalytic subunit of PRC2, functions as a histone methyltransferase, catalyzing methylation of lysine 27 on histone H3 (H3K27), leading to gene silencing through H3K27me3. EED facilitates interaction with chromatin, while SUZ12 is essential for the integrity of the complex [76,77]. Alterations in any of these subunits can disrupt the functional equilibrium of the complex and contribute to abnormal oncogenic phenotypes (Figure 3) [74,75].

Gain-of-function somatic mutations in EZH2 suggest an oncogenic role in non-Hodgkin lymphoma [78]. Aberrant expression of wild-type EZH2 has similarly been implicated in various adult cancers [79,80,81]. However, EZH2 loss-of-function mutations have been observed in myeloid malignancies [82] and T-cell ALL [83]. Haploinsufficiency for PRC2 components has also been linked to increasingly aggressive clinical behavior in pediatric AML [84], while EED or SUZ12 inactivation is recurrently observed in sporadic, Neurofibromatosis type 1 (NF1)-associated, and radiotherapy-associated Malignant Peripheral Nerve Sheath Tumor (MPNST) [85].

Aside from the direct mutation or altered expression of PRC2 components, many pediatric cancers exhibit indirect disruption of PRC2 function. Oncogenic histone H3 K27M mutation in incurable pediatric DMG leads to dominant-negative inhibition of PRC2 activity with global loss of H3K27me3 [12]. Similarly, posterior fossa group A ependymomas aberrantly express an open reading frame encoding the protein EZHIP [86], which directly interacts with and inhibits EZH2 [87], leading to altered chromatin regulation and organization [88]. Cancers without obvious mutational drivers of EZH2 disruption can nonetheless display a striking enrichment of epigenetic alterations specifically over PRC2 regulatory targets, such as in the convergent epigenetic signature seen across multiple cytogenetic subtypes of pre-B-cell lymphoblastic leukemia [89]. Altered PRC2 regulation is widespread in cancer [5], highlighting the potentially therapeutic utility of targeting PRC2 components.

The oncogenic dependence of cancer cells on EZH2 has sparked interest in it as a therapeutic target, leading to the development of several inhibitors. Initially, 3-Deazaneplanocin (DZNep) was used, which disrupts the methionine cycle by impeding S-adenosyl-L-homocysteine hydrolase, causing a broad inhibition of SAM-dependent histone methyltransferases albeit not specific to EZH2 [90]. More targeted efforts have produced potent inhibitors like EPZ005687 and GSK126, which selectively block EZH2 and halt cell growth in Diffuse Large B-Cell Lymphoma (DLBCL) [91]. EI1 further improved selectivity by inhibiting both wild-type and mutant EZH2 [92], and UNC1999 was developed as an orally bioavailable EZH2/EZH1 inhibitor [93]. Of particular note, EPZ-6438, also known as Tazemetostat, combined excellent inhibitory potency and oral bioavailability and gained ground in the therapeutic approach to rhabdoid tumors [94]. Rhabdoid tumors are driven by biallelic *SMARCB1* inactivation; this loss disrupts the SWI/SNF chromatin remodeling complex, a natural antagonist of PRC2, leading to aberrant PRC2 activation [95]. In the NCI-COG Pediatric MATCH Phase II subprotocol C, tazemetostat, despite not meeting its primary efficacy endpoint, demonstrated potential as a disease stabilizer in 20 young patients with highly aggressive tumors carrying mutations in members of the SWI/SNF complex or EZH2 (NCT03213665) [96]. Additionally, tazemetostat has been suggested as a post-chemotherapy maintenance therapy for rhabdoid tumors [97].

In addition to these promising results, Tazemetostat has received FDA approval for the treatment of aggressive adult cancers, including follicular lymphoma (NCT01897571) [98] and epithelioid sarcoma, with the latter applied for patients older than 16 years (NCT02601950) [99].

Significant preclinical data support the potential of EZH2 inhibition in pediatric cancer treatment. EZH2 inhibition has been suggested to enhance sensitivity to radiation in ATRT [100], and when combined with Bromodomain protein 4 (BRD4), inhibition has shown therapeutic benefits both in vitro and in vivo in pediatric rhabdoid tumors [101]. EPZ6438 has been demonstrated to impede cell proliferation and survival in patient-derived DMG cell lines, linked to *p16* induction [102]. Dual targeting of BMI1, a PRC1 component, and EZH2 has also hindered glioma stem cell growth both in vitro and in vivo, constraining tumor heterogeneity [103]. EZH2 inhibitors have demonstrated effectiveness in specific subtypes of pediatric AML overexpressing EZH2 [104], and in primary pediatric B-cell and T-cell ALL samples [105].

The complex functions of PRC2 suggest that targeting its non-catalytic regulatory subunits, such as EED, may lead to more effective inhibition [106]. Disruption of the EZH2/EED complex in MLL-AF9 leukemia cells resulted in growth arrest and decreased viability [107]. The EED protein–protein interaction inhibitor A-395 exhibited similar effects to EZH2 inhibitors while remaining effective against resistant cell lines [108]. This innovative strategy aims to destabilize the complex and reduce its downstream effects in cancer development, showing particular promise in cell lines resistant to existing PRC2 inhibitors.

#### 2.2.2. Histone Methylation

The NSD protein lysine methyltransferase (KMT) family encompasses three members, NSD1, NSD2, and NSD3, which mono- and di-methylate H3K36, producing H3K36me1 and H3K36me2. H3K36me2 also regulates H3K36me3 levels by acting as a substrate for tri-methylating enzymes like SET domain containing 2 (SETD2) [109]. Mutations in NSD1 are linked to Beckwith-Wiedemann and Sotos syndromes, both associated with increased cancer susceptibility [110]. The t(5;11)(q35;p15.5) translocation, resulting in the NUP98/NSD1 fusion gene, has been recognized as a frequent event in pediatric cytogenetically normal AML with prognostic significance [111]. This fusion gene has been found to colocalize H3K36 methylation with H3/H4 acetylation at regulatory elements, leading to sustained Hox-A expression and myeloid progenitor maintenance [112]. Moreover, NSD1 hypermethylation is prevalent in neuroblastoma and glioma [113], highlighting its role as a tumor suppressor gene, while NSD2 mutations are frequently observed in pediatric ETV6-RUNX1 ALL [114].

Genetic alterations involving the histone methyltransferase gene *KMT2A*, also known as *MLL* or mixed lineage leukemia, highlight its central role in oncogenesis. Translocations involving chromosome band 11q23, where *MLL* is located, are observed in the majority of infant ALL cases, as well as in cases of pediatric and adult ALL and AML [115]. KMT2A functions as a H3K4 methyltransferase, but its methyltransferase activity is commonly disrupted in the resulting fusion genes, suggesting that its transforming effect occurs via altered recruitment of epigenetic regulators [116,117]. Considerable attention has focused on how MLL drives oncogenic transformation and the roles of specific translocation partners [118,119]. Aberrant expression of HOXA proteins and the upregulation of HOX cofactor MEIS1 are critical downstream effects in MLL-rearranged AML [120], leading to aberrant differentiation and lineage plasticity [117].

Various strategies for targeting altered epigenetic regulation in MLL-rearranged leukemia have been investigated, centering around the interaction of MLL with chromatin-associated complexes. MEN1 has been recognized as critical in maintaining the leukemic phenotype through its interaction with MLL. The selective Menin–MLL inhibitor, VTP50469, has demonstrated efficacy in disrupting this interaction, resulting in decreased expression of MLL target genes and improved survival in mouse models [121]. Within the MLL interactome, the histone methyltransferase DOT1L is heavily recruited to MLL target genes, leading to H3K27 methylation at loci such as HOXA9 and MEIS1, leading in turn, to enhanced transcription and promoting leukemogenesis. Targeting DOT1L with inhibitors like pinometostat (EPZ-5676) has shown promise in MLL-rearranged leukemia xenograft models, achieving significant tumor regression [122], while in the clinical setting, although well tolerated, pinometostat has shown modest clinical activity in adult leukemia (NCT01684150) [123].

Histone 3 lysine 9 (H3K9) di- and tri-methylation (me2/me3) are generally associated with silenced transcription and heterochromatin [124]. Mammalian H3K9 methyltransferases are divided into the suppressor of variegation (SUV) family, comprising suppressor of variegation 3-9 homologue 1 (SUV39H1) SUV39H2, SET domain bifurcated 1 (SETDB1), SETDB2, G9a, and G9a-like protein (GLP) [125], and the PR domain containing (PRDM) family [124,126].

Alterations in H3K9 methylation, often due to overexpression of H3K9 methyltransferases leading to increased H3K9 methylation at tumor suppressor gene promoters, have been shown to play an important role in cancer [124]. H3K9me3 has been found to be enriched in both low- and high-grade pediatric astrocytoma, correlating with reduced patient survival [127]. SUV39H1 expression was found to be increased in pilocytic astrocytomas, while SETDB1 expression has been found to be upregulated in pediatric high-grade gliomas (pHGGs), including diffuse astrocytomas and glioblastomas, indicating that SUV39H1 may establish H3K9me3 in grade I tumors, while SETDB1 may establish the same mark in high-grade tumors [127,128]. Silencing H3K9 methyltransferases using shRNAs was shown to decrease both H3.3K27M-mutant and H3.3G34R-mutant pHGG cell viability [126]. Furthermore, increased H3K9me3 in H3K27M gliomas may compensate for the loss of H3K27me2/3 silencing, as higher H3K9me3 levels are found in regions with lower H3K27me2/3. Notably, astrocytes expressing the double mutant H3.3K9M/K27M prevent the global reduction in H3K27me3 [126,129].

In neuroblastoma, G9a and GLP were found to have a positive correlation with MYCN expression, and increased H3K9me2 and H3K27me3 at the CXCL9, CXCL10, and CXCL11 chemokine cluster indicated the potential role of G9a, GLP, and EZH2 in maintaining a ‘cold’ tumor microenvironment [130]. In medulloblastoma, G9a was found to silence USP37, and G9a inhibition suppressed growth [131]. In alveolar rhabdomyosarcoma (ARMS), increased SUV39H1 levels were found to impair MyoD, blocking myogenic differentiation and growth arrest [132]. Additionally, SETDB2 overexpression observed both in vitro and in vivo in pre-BCR+ ALL was found to suppress expression of the cell-cycle inhibitor CDKN2C through H3K9me3 in E2A-PBX1+ B-ALL [133].

While no H3K9 methyltransferase inhibitors have moved to clinical trials, several have shown promise in preclinical models of childhood cancer. Chaetocin, a natural product with inhibitory activity against SUV39H1, decreased the proliferation and viability of astrocytoma and oncohistone-mutant pHGG cells [126,127,134], although it was not selective [135]. OTS186935, a more potent SUV39H2 inhibitor, has also been shown to reduce the proliferation of oncohistone-mutant pHGG [126,136,137]. BIX01294 and UNC0638, both G9a/GLP inhibitors, suppressed astrocytoma and neuroblastoma cell growth while increasing IFN-y-induced expression of CXCL10 mRNA in MYCN-amplified neuroblastoma [127,130,138]. E67, a modified version of BIX01294 and UNC0638, decreased medulloblastoma cell proliferation, but UNC-0638 was more effective [131,139]. UNC0642, a more potent analog of UNC0638 with better in vivo pharmacokinetics, inhibited growth in several pediatric cancers, including MYCN-amplified neuroblastoma [140], Ewing sarcoma [141], and ARMS [142,143].

#### 2.2.3. Histone Demethylation

Alterations involving histone demethylases are also common across cancer. For instance, H3K27 demethylase UTX (KDM6A) is frequently mutated in several cancers, including solid and hematologic malignancies [144]. KDM6B plays a key role in hematopoietic stem cell self-renewal [145], while KDM4/JMJD2, H3K9-, and H3K36-specific demethylases are essential for maintaining hematopoietic stem cell populations [146]. Overall, histone demethylases function in a context-dependent manner and can promote or oppose tumorigenesis, as indicated by conflicting signatures of the H3K27 demethylases JMJD3 and UTX in T cell ALL [147]. Interestingly, the oncometabolite 2-HG produced by IDH-mutant tumors has been shown to inhibit KDM5 histone lysine demethylation, contributing to its transforming influence in IDH-mutant leukemia and glioma [148].

KDM4C, an H3K9 demethylase, contributes to the epigenetic reprogramming of AML by removing repressive H3K9me2 repressive marks and favoring activating histone acetylation [149]. Similarly, KDM2B was upregulated in Ewing sarcoma and correlated with worse prognosis [150], and KDM3A was also found to promote the migration in vitro and metastasis in vivo of Ewing sarcoma [151].

Similarly to other enzyme families, there is ongoing interest in developing molecules targeting histone demethylases. KDM inhibitors have shown preclinical potential in suppressing tumor growth in Ewing sarcoma [152], while several KDM inhibitors have been applied to AML with promising results [153,154]. Notably, the introduction of LSD1/KDM1A inhibition (targeting a H3K4me1/2 and H3K9me1/2 demethylase) in a melanoma mouse model resulted in IFN signaling induction and sensitization to checkpoint inhibition, thus stimulating tumor immunogenicity [155].

Several phase I clinical trials have evaluated KDM inhibition in cancer. For example, LSD1 inhibitors have been explored in the context of AML and Ewing sarcoma (NCT02712905) [156]. However, the translation of their promising preclinical results into clinical outcomes requires further investigation, particularly for pediatric cancer indications.

#### 2.2.4. Histone Acetylation and Deacetylation

Histone acetylation involves the transfer of an acetyl group from acetyl-coenzyme A (acetyl-CoA) to specific lysine side chains within the basic N-terminal tail region of a histone. Histone acetyltransferases (HATs) facilitate acetylation, and histone deacetylases (HDACs) carry out deacetylation [157,158]. Typically, histone acetylation is linked with transcriptional activation, promoting a more accessible chromatin structure [159].

Mutations in proteins of the HAT families have been widely associated with cancer [160]. Somatic mutations of *p300* and *CBP* are prevalent across a wide spectrum of tumors, including both solid and hematological malignancies [161]. Moreover, members of the HAT family have been identified among common translocation partners of MLL in MLL-rearranged acute leukemia [162]. A growth dependence on EP300 has also been observed in high-risk pediatric neuroblastoma, with EP300 degradation resulting in loss of MYCN and subsequent cell death [163].

HDACs also play a significant role in pediatric cancer. HDAC8 has been identified as a potential driver of tumor growth in neuroblastoma, with its expression correlating with disease progression [164]. Interestingly, the overexpression of HDAC7 and HDAC9 has been linked with poor prognosis in childhood ALL [165]. Additionally, HDAC inhibition was shown to have immune-regulatory properties in diverse immune cell populations after allogeneic hematopoietic cell transplantation [166].

Pharmacologic targeting of HDACs has been a prominent strategy in targeting cancer-associated epigenetic alterations. Therefore, suberoylanilide hydroxamic acid (SAHA or vorinostat) and depsipeptide (romidepsin) have received FDA approval for treating cutaneous T-cell lymphoma in adults [167,168], with romidepsin and belinostat also approved for peripheral T-cell lymphomas [169,170].

Safety and tolerability have been explored across a panel of HDAC inhibitors, with their potential to sensitize tumor cells to radiation paving the way for their integration into the treatment of cancers traditionally managed with radiation [171]. Due to preclinical drug screening showing the efficacy of HDAC inhibitors against histone H3 K27M DMG cell lines [172], panobinostat was advanced to clinical trials for recurrent/progressive DMG, but was limited by poor brain penetration (PBTC-047, PNOC015) [173,174]. Similarly, in a phase I/II trial, vorinostat demonstrated a favorable toxicity profile in children with newly diagnosed DMG, but failed to translate its preclinical efficacy in sensitizing glioma cells to radiation into clinical benefit (ACNS0927) [175]. A phase I trial demonstrated good tolerability for entinostat in pediatric populations with Recurrent or Refractory Solid Tumors, albeit with no encouraging results in terms of survival (NCT02780804) [176]. Similar findings were observed in a phase I trial of panobinostat in relapsed and refractory pediatric hematologic malignancies (NCT01321346) [177], and pracinostat in children with refractory solid tumors (NCT01184274) [178]. A phase I/II trial investigated the feasibility of escalating vorinostat doses in pediatric populations with a variety of relapsed tumors, including both solid and hematologic tumors, yielding improved responses and favorable toxicity (NCT01422499) [179].

The potential of HDACi to reprogram cancer cells by reversing treatment resistance or sensitizing them to conventional chemotherapy and newer targeted therapies has prompted investigations into combining HDACi with other compounds. For instance, incorporating vorinostat along with isotretinoin in intensive chemotherapy for young patients with embryonal CNS tumors has demonstrated favorable survival outcomes (PBTC-026) [180]. Additionally, in a randomized phase II trial, the combination of Meta-iodobenzylguanidine (MIBG) with vorinostat has shown a superior response rate in patients with relapsed or refractory neuroblastoma compared to irinotecan or vincristine (NCT02035137) [181].

Despite encouraging preclinical findings and positive safety profiles, these strategies are yet to be effectively translated for pediatric indications. Current investigations are shifting from single-agent uses of epigenetic drugs to utilizing these as adjuncts to chemotherapy or immunotherapy. As discussed previously, HDACi’s combination with DNA-hypomethylating agents such as decitabine has demonstrated promising results in de-repressing silenced genes and inducing immune activation, potentially opening up new avenues for clinical investigation.

### 2.3. Readers

While epigenetic writers and erasers add or remove groups post-translationally, chromatin readers like bromodomains recognize and bind to these marks, recruiting proteins and protein complexes that alter gene expression and chromatin structure. Bromodomains, such as those in the bromodomain and extra terminal (BET) family (BRD2, BRD3, BRD4, and BRDT), bind to acetylated histone tails [182,183]. BRD4, the most frequently studied bromodomain in cancer research, recruits transcription factors essential for RNA polymerase II-dependent transcription [184,185]. When dysregulated, BRD4 can play a role in upregulating oncogenes and disrupting the cell cycle, as seen in nuclear protein in testis (NUT) midline carcinoma (NMC), where a BRD4-NUT fusion recruits histone acetyl transferases, subsequently promoting MYC upregulation and inhibiting cell differentiation [186,187].

BET inhibitors, such as BI 894999 and JQ1, have shown promise in dislodging BRD-NUT from chromatin and inducing anti-proliferative effects [188,189]. One key mechanism downstream of BET inhibition in cancer occurs via downregulation of the oncogenic transcription factor MYC [190,191]. MYC is activated and dysregulated in a variety of childhood cancers, including neuroblastoma, Wilms tumor, ATRT, and medulloblastoma [101,192,193]. In preclinical studies of Wilms tumor, the bivalent BRD4 inhibitor AZD5153 led to reduced MYC levels in cell lines derived from anaplastic and non-anaplastic Wilms tumor, and inhibited tumor growth in patient-derived xenografts [194].

Preclinical studies of JQ1 in medulloblastoma have shown increased survival and anti-proliferative effects, particularly in MYC-expressing cells [195]. JQ1 also showed benefit in combination with an EZH2 inhibitor in ATRT models [101]. However, clinical trials for BET inhibitors in pediatric cancers remain limited, with only one ongoing trial (NCT03936465), including pediatric solid tumors or lymphoma in the first arm, and pediatric brain tumors or tumors that have metastasized to the brain in the second arm.

**Figure 4:**
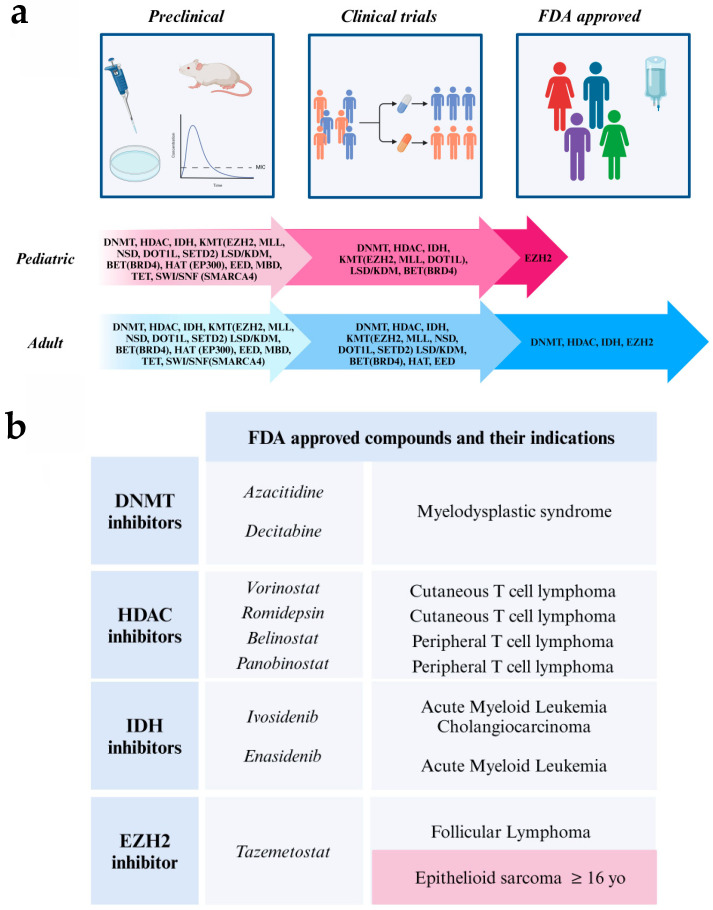
(**a**) Advancements in epigenetic drug development—adult and pediatric indications. (**a**) For adult oncology indications, FDA-approved drugs inhibit four major targets: DNMTs, EZH2, HDACs, and mutant IDH. In pediatric oncology, tazemetostat, an EZH2 inhibitor, is the only FDA-approved compound, specifically for the treatment of epithelioid sarcoma in patients aged 16 years and older. Despite ongoing interest in epigenetic regulation and efforts to target a broader range of epigenetic marks preclinically, few compounds successfully progress through clinical trials to FDA approval. This challenge is even more pronounced in pediatric oncology. (**b**) FDA-approved epigenetic drugs and their indications. Blue boxes indicate approvals for adult cancers, while the pink box highlights the approval of tazemetostat for epithelioid sarcoma in patients aged 16 years and older.

As discussed above, significant progress has been made in epigenetic treatments. Many epigenetic targets are associated with cancer, providing opportunities to develop and implement therapeutic strategies. Preclinical efforts are exploring various targets and mechanisms, with only a few advancing to clinical trials and even fewer receiving FDA approval to date. This landscape is even more limited for pediatric cancer, despite the central role of altered epigenetic drivers in these cancers (Figure 4a,b).

## 3. Epigenetic Variability

**Figure 5:**
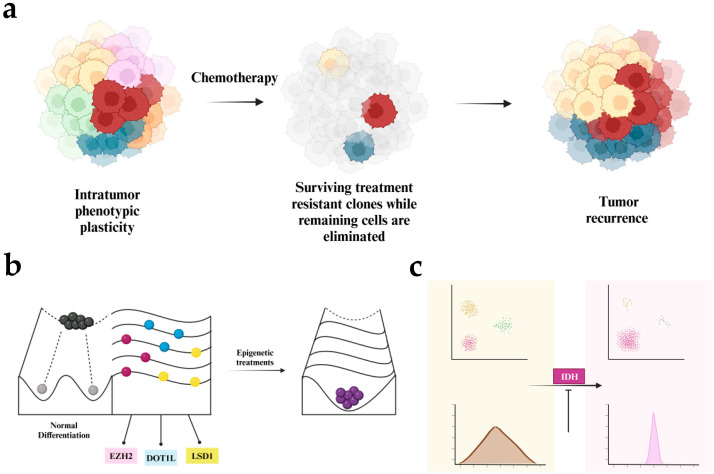
Role of epigenetic plasticity in cancer. (**a**) Phenotypic plasticity as a driver of tumor recurrence. The primary tumor contains a mixture of cell states, exhibiting various states of phenotypic plasticity enabled by the cancer epigenetic landscape. Epigenetic variability driving phenotypic plasticity can enable the survival and selection of resistant cells. (**b**) Epigenetic landscapes in normal development and cancer. During normal development, differentiating cells can be conceptualized as marbles rolling down a hill, with barriers in the epigenetic landscape preventing them from de-differentiating (rolling backwards) or trans-differentiating (rolling to the side), and guiding them toward a defined, low-entropy cell state. In cancer, the epigenetic landscape is flat, with a lower energy cost for cell state transitions, allowing cancer cells to shift stochastically between states. Epigenetic modifiers such as EZH2, DOT1L, and LSD1 establish and regulate the epigenetic landscape. Epigenetic therapies could potentially restore this landscape, lowering epigenetic entropy in cancer. (**c**) Oligodendroglioma as an illustration of this concept. IDH1 mutant oligodendroglioma is characterized by diverse subpopulations. Inhibition of mutant IDH1, a modulator of the aberrant epigenetic landscape, can push cells toward specific fates, eliminating some populations while promoting others.

An added layer of complexity in our understanding of epigenetic dysregulation in cancer has been the recent appreciation of epigenetic variability as a determinant of cancer cell heterogeneity and plasticity, and as a driving force in tumor evolution. The emergence of epigenetic stochasticity, or non-deterministic changes in epigenetic marks giving rise to epigenetic variation, can underlie the phenotypic plasticity of cancer cells and allow for the selection of cellular traits that promote growth and survival despite a changing environment and therapeutic stress (Figure 5a) [196]. Adapting the Waddington landscape of development, which represents differentiation as a marble rolling down a hill to a defined low-energy state, we can conceptualize epigenetic dysregulation in cancer as ‘flattening’ the potential energy landscape, thus eroding the energy barriers to de-differentiation or trans-differentiation and removing the attractor toward normal differentiation [3]. This illustrates how epigenetic instability and increased epigenetic entropy can support the emergence of malignant cells and subsequent plasticity between various cell states in cancer (Figure 5b). Epigenetic instability is supported as a general mechanism in cancer by the discovery of increased DNA methylation variation localizing to specific genomic domains across a variety of cancers, including adult colon, lung, breast, thyroid, and hematological malignancies, and pediatric Wilms tumor and hematologic malignancies [19]. Recent work identified a higher degree of DNA methylation heterogeneity in chronic lymphocytic leukemia, resulting from seemingly stochastic variation in methylation patterns; importantly, disordered methylation was associated with adverse clinical outcomes [197]. Further, a DNA methylation analysis of paired diagnostic and relapse samples in diffuse large B-cell lymphoma showed that increased methylation heterogeneity at diagnosis was predictive of relapse [198]. A DNA methylation analysis in AML showed that epigenetic variation, as assessed by epigenetic allele burden, was related to inferior clinical outcomes [199]. In Ewing sarcoma, a DNA methylation analysis using reduced-representation bisulfite sequencing revealed widespread intratumor epigenetic heterogeneity, particularly in patients with metastatic disease [200].

Since pediatric cancers are largely epigenetically driven, understanding and targeting mechanisms of epigenetic variability are critically important. Epigenetically determined phenotypic plasticity can underlie complex changes, such as epithelial–mesenchymal transition (EMT), drug resistance, the emergence of aberrant differentiation, and metabolic states. Recent work has been aimed at capturing the dynamic epigenetic landscape of cancer cells and understanding the epigenetic underpinnings of cancer cell plasticity. This has relied on the application of novel mathematical methods to model the nondeterministic nature of epigenetic regulation, and to quantify epigenetic stochasticity and map its targets genome-wide. The application of pharmacologic strategies to constrain epigenetic variability would represent a novel approach to epigenetic therapy, aiming to limit the emergence of resistant subpopulations, or alter the epigenetic landscape in a way that favors therapeutic differentiation.

Significant methylation discordance has been documented in pre-B ALL, and has been shown to localize to key genomic regions that inform the leukemic phenotype. This underscores the importance of methylation stochasticity as a critical epigenetic regulator of disease development even in the context of the most common pediatric cancer, traditionally associated with specific genetic alterations [89]. Similarly, DMG, a tumor with a low mutation burden, was also shown to exhibit methylation discordance, mapped to key regulatory pathways, related to pluripotency and developmental reprogramming. Decitabine was found to constrain this methylation discordance in specific regulatory regions, suggesting that this offers another perspective to target tumor evolution and plasticity for therapeutic benefit [59].

Epigenetic variability can also drive the phenotypic plasticity observed in cancer, suggesting that restraining this variability could more effectively capture the cells in defined cell fates. In IDH mutant oligodendroglioma, a single-cell RNA-sequencing analysis revealed two dominant glial programs, astrocyte-like and oligodendrocyte-like, along with a rare subpopulation of undifferentiated cells linked to a neural stem cell signature [201]. Intervention with an IDH inhibitor drove oligodendroglioma cells toward astrocytic differentiation, depleting stem-like cells and diminishing cell proliferation (Figure 5c) [202]. Similarly, IDH2 inhibition in IDH2 mutant leukemia cells elicited a leukemic cell differentiation response, suggesting another avenue that could reverse the differentiation block observed in AML [203]. Additionally, corin, a bifunctional LSD1 and HDAC inhibitor, was shown to induce differentiation in DMG both in vitro and in xenograft models, while also inhibiting cell proliferation. This demonstrates that targeting multiple epigenetic modifications can confer synergistic therapeutic effects not observed when applying a single class of inhibitors [204].

Methylation stochasticity underlies the heterogeneity and plasticity that serve as hallmarks of cancer. Mapping the targets of the destabilized epigenome can aid in dissecting epigenetically altered drivers of malignant phenotypes. Mapping the dynamic epigenetic landscape in a way that encapsulates stochastic variation can elucidate new targets to combat cancer plasticity and resistance.

## 4. Cancer Epigenetics and Immunotherapy

Among the major determinants of tumor immunogenicity is mutational burden, such that cancers with mutations in machinery involved in DNA repair, such as mismatch repair (MMR) genes, are more immunogenic. A high mutational burden fuels the ‘cancer immunity cycle,’ in which dendritic cells (DCs) capture antigens from the tumor and present them to naïve T cells, priming the T cells to invade the tumor and initiate cancer cell killing [205]. Pediatric cancers are especially adept at evading immune surveillance. Cancers with a high mutational burden are rare in childhood, making childhood cancers, on average, significantly less immunogenic than adult cancers [206].

Although pediatric cancers are rarely hypermutated, there is a range of immunogenicity in pediatric cancers. At one end of the spectrum lie pediatric High-Grade Gliomas (pHGGs), with a high mutational burden from mutations in genes such as *POLE*, *POLD1*, or *MLH1*. These pHGGs are of the histone wild type and have been shown to have a significant percentage of infiltrating CD8+ T cells. On the other end of the spectrum are histone mutant gliomas, such as DMGs, which, when compared to their histone wild-type counterparts, have been shown to be immunologically cold, with few tumor-infiltrating lymphocytes (TILs) [207]. Although the identification of targetable, tumor-specific antigens has been a challenge, there have been successes, highlighting the potential of immunotherapy for pediatric brain tumors. High expression of the disialoganglioside, GD2, in DMG has led to promising results from GD2-directed CAR-T-cell therapy in mouse models and ongoing clinical trials (NCT04196413) [208]. Similarly, the over-expression of B7-H3 (CD276) in central nervous system tumors has led to trials of intraventricular B7-H3 CAR-T cells (NCT04185038) [209].

Given that pediatric cancers are rarely hypermutated, there is a growing interest in finding ways to make them more immunogenic, and pharmacologic modulation of the cancer epigenome has emerged as a promising avenue for this purpose. Recent evidence from nine adult cancer cell lines has shown that BRD4 inhibitors can be used to convert MMR-proficient (pMMR) tumors into MMR-deficient (dMMR) tumors by decreasing the expression level of key MMR genes. As mentioned above, tumors with MMR deficiency are more immunogenic due to increased tumor mutational burden and neoantigen expression. Interestingly, it was found that tumors maintain a dMMR phenotype even when they develop resistance to the applied BRD4i [210]. Therefore, BRD4i may prove to be a potential avenue for increasing the responsiveness of pediatric cancers to Immune Checkpoint Blockade (ICB) therapy.

**Figure 6:**
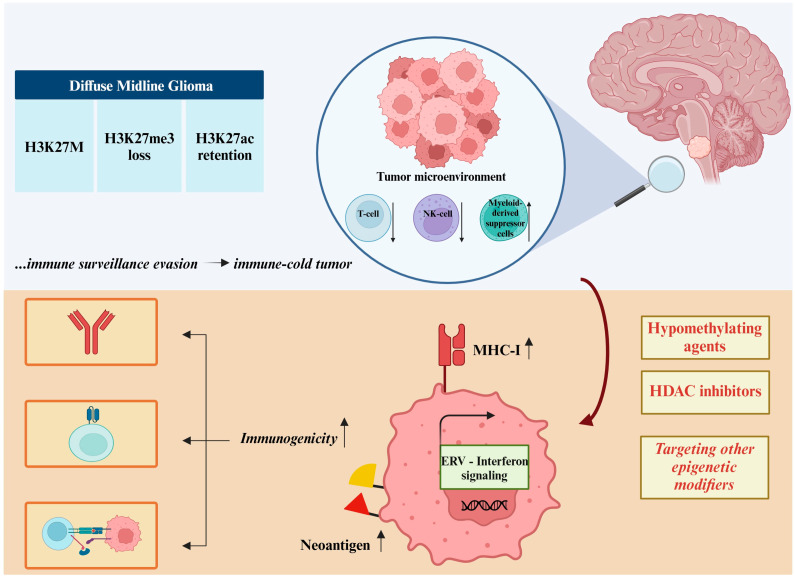
Immunomodulatory effects of epigenetic therapies—the example of Diffuse Midline Glioma (DMG). DMG is driven by the mutant histone H3K27M, accompanied by the loss of H3K27me3 and the retention of H3K27ac. DMG is an ‘immune-cold’ cancer, characterized by minimal infiltration of T and NK cells, with myeloid-derived suppressor cells prevailing. Modulating DMG with hypomethylating agents, HDAC inhibitors, or novel epigenetic approaches can promote immune induction. Specifically, cancer cells enhance MHC-I expression, neoantigen presentation, and ERV, STING, and interferon signaling. Therapy could promote an ‘immune-hot’ state, enabling the application of immunotherapy strategies such as antibodies, CAR T-cells, and checkpoint inhibitors.

Additionally, efforts over the last decade have utilized DNMTis, such as decitabine and azacytidine, and HDAC inhibitors to make cancer cells more immunogenic. These agents achieve this by inducing viral mimicry through the dsRNA sensing pathway, by increasing the expression of endogenous retroviruses (ERVs) and Cancer Testis Antigens (CTAs), and by increasing the expression of MHC Class I, thus enhancing the cell’s capacity for antigen presentation [59,211,212,213]. Additionally, CTAs upregulated by epigenetic modulating agents may already be the targets of existing immunotherapy approaches. These approaches can be applied to traditionally immune-cold tumors, such as DMG, utilizing epigenetic modulation to shift the tumor’s immune-silent profile to an immune-hot phenotype, thereby opening the door for effective immunotherapy strategies (Figure 6). Decitabine has also been shown to upregulate the expression of CTAs and MHC Class I/II molecules, thereby enhancing the susceptibility of rhabdomyosarcoma and Ewing sarcoma cell lines to cytotoxic T lymphocyte (CTL)-mediated killing [214]. In the clinical setting, decitabine has been shown to upregulate the expression of the CTA, PRAME, which is the target of an existing immunotherapy, IMC-F106C, in clinical trials (NCT04262466) [59]. This suggests that hypomethylating and deacetylating agents could be employed as sensitizers to ICB or another form of immunotherapy.

Another barrier to the success of cancer immunotherapy is the development of acquired resistance. For example, a study comparing matched pretreatment and ICB-resistant adult Non-Small Cell Lung Cancer (NSCLC) demonstrated that resistant tumors have a loss in neoantigens that previously elicited strong T-cell clonal expansion [215]. While acquired resistance to ICB is poorly understood, evidence in adult pancreatic ductal carcinoma (PDAC) suggests that epigenetic and transcriptional alterations of gene expression are responsible for resistance. In PDAC specifically, investigators found that ICB resistance resulted in a silencing of the gene interferon regulatory factor-6 (*Irf6*). *Irf6* silencing made the tumor cells resistant to ICB because they were no longer susceptible to T-cell killing by TNF-alpha. The restoration of *Irf6* renewed the sensitivity of PDAC to ICB [216]. Previous studies focusing on DMG have shown that DNMTis are capable of upregulating the expression of interferon genes and may have utility in reversing ICB resistance [59]. Furthermore, investigations on ICB-resistant adult Merckel Cell Cancer have revealed that resistant tumors develop a transcriptionally dependent downregulation of MHC Class I, which was shown to be reversible with 5-azacitidine [217]. It has also been shown that MHC Class I expression is regulated by PRC2 components, suggesting that treatment with an EED inhibitor may be effective in increasing tumor immunogenicity or reversing ICB resistance [218]. Although many of the presented findings were discovered in the adult setting, it is likely that the pharmacological modulation of the epigenome could be useful in increasing the baseline immunogenicity of cancers and reversing their acquired resistance to ICB, even in pediatric patients.

While it is likely that the abovementioned techniques will be useful in enhancing the immunogenicity of pediatric cancers, there is evidence that there may be further explanations for why pediatric cancers are less responsive to ICB compared to their adult counterparts. To begin, there is some evidence to suggest that robust anticancer immune responses can be generated in pediatric cancers, such as ALL, despite their relatively low mutation burden [219]. Such evidence suggests that the reduced responsiveness of pediatric cancers to immunotherapy may be due to innate differences in the child/adolescent immune system compared to the adult immune system. Indeed, studies in young vs. old tumor-bearing mice have shown that young patients’ CD8+ T cells are often more potent but become exhausted more rapidly than adult CD8+ T cells. Additionally, CD8+ T cells are also primed to attain a terminally differentiated state in a young immunological environment [220]. It is worth conducting further investigations to see whether epigenetic-targeted therapies can reverse such differentiation and make CD8+ T cells more plastic and less easily exhausted.

## 5. Combination Therapies

A different approach could incorporate combination therapies. Leveraging the synergistic effects of epigenetic therapies with other targeted therapeutics, conventional chemotherapy, or immunotherapy offers another avenue for targeting pediatric cancer.

On the subject of combining multiple epigenetic therapies, the strategy of combining DNA-hypomethylating drugs with HDAC inhibitors has been well-studied in a variety of adult solid and hematologic cancers [212,213]. For instance, strong preclinical evidence supports the synergism of HDAC inhibitors and decitabine in the context of DLBCL [221]. Interestingly, the combination of decitabine and vorinostat has been shown to improve quality of life and induce stabilization of marrow disease in two pediatric patients with secondary MDS/AML concurrent with solid tumor relapse [222]. Consistent synergy in halting cell proliferation has also been observed with EPZ-5676, a DOT1L inhibitor, and DNMT inhibitors in MLL-rearranged leukemia cell lines [223]. Furthermore, azacytidine combined with BRD4 inhibition has shown potential synergism in inducing apoptosis, possibly through the activation of the DNA damage response (DDR) pathway, in myeloid leukemia cell lines [224]. The combination of epigenetic therapies has also been implicated in altering the tumor microenvironment. Notably, in high-risk MYCN-amplified neuroblastoma, H3K9 euchromatic histone lysine methyltransferase (EHMT) inhibitors in combination with EZH2 inhibitors exert immunomodulatory effects by inducing strong IFN-γ downstream responses [130]. Additionally, corin, a bifunctional LSD1 and HDAC inhibitor, not only suppressed tumor growth, but also promoted a cellular differentiation phenotype [204].

Epigenetic mechanisms can drive treatment resistance, suggesting that targeting the epigenetic machinery can enhance conventional chemotherapy or restore the drug sensitivity of resistant cells [225]. Notably, the pretreatment of patient-derived relapsed childhood B-cell ALL samples with decitabine and vorinostat has been shown to reverse resistance to prednisolone [226]. As noted previously, in clinical trials of relapsed B-cell ALL, this strategy offered promise but was associated with a high rate of infectious toxicities (NCT01483690) [55]. A phase I trial evaluating the addition of decitabine and vorinostat to the cytotoxic backbone of the treatment of pediatric relapsed/refractory or therapy-related myeloid malignancies showed good tolerability and effectiveness, particularly in patients with epigenetic modifications (NCT03263936) [52]. Decitabine has also been used as a salvage therapeutic option in combination with Cisplatin, Cytarabine, and Dexamethasone for relapsed or refractory DLBCL after second-line treatment failure, yielding encouraging response outcomes (NCT03579082) [227]. Further research will be needed to optimize rational combinations of epigenetic and conventional therapies. For instance, EPINUC, a tool that allows for detailed analysis of histone modifications at the single-molecule level, and single-cell mass spectrometry approaches to detect histone modifications highlight the need for more detailed studies of co-occurring epigenetic alterations [228,229]. Understanding these patterns can help to clarify the rational design of epigenetic combination therapies.

Combinations of epigenetic therapies with targeted therapies have also been investigated. CUDC-907, a dual PI3K and HDAC inhibitor, has been shown to suppress the growth of MYC-driven neuroblastoma and medulloblastoma and to exhibit radiosensitizing properties in DMG models by inhibiting radiation-induced DDR pathways [230,231,232]. Furthermore, decitabine has been shown to enhance DLBCL dependence on Bcl-2, sensitizing both in vivo and in vitro models to the Bcl-2 inhibitor, venetoclax [233]. The approval of the combination of azacitidine with venetoclax in adult AML patients ineligible for intensive chemotherapy has led to the evaluation of its safety in pediatric patients with AML (NCT02993523) [234,235]. Additionally, this combination has shown potential as a salvage treatment for young patients with multiply relapsed and refractory leukemias [236].

Additionally, targeting the epigenetic machinery holds significant immunomodulatory potential. Epigenetic therapies have been shown to induce MHC-I expression, reactivate endogenous retroviral elements, trigger neoantigen expression, and upregulate interferon signaling in cancer cells. These effects make cancer cells more susceptible to immune targeting and pave the way for effective immunotherapy combinations [237].

Combination strategies are in the early stages of development, and more research is needed to determine optimal dosing and sequencing pattern of epigenetic interventions as part of multi-agent treatment regimens, focusing not only on cytotoxic efficacy, but also on the prevention of resistance/relapse and limiting toxicities [238].

Another important area of future research in epigenetic therapeutics will involve selecting the optimal subset of patients for specific therapies. The concept of using methylation or other epigenetic signatures to guide treatment selection can lead to the establishment of epigenetic biomarkers, which could potentially stratify patients and optimize their treatment benefits.

## 6. Discussion

In this review, we attempted to summarize existing epigenetic therapies and emerging perspectives on the application of epigenetic therapeutics to pediatric cancer. Overall, childhood cancer has a lower mutational burden, with epigenetic alterations often taking on central roles in tumor initiation and progression.

Interestingly, despite the diversity of cancer drivers across the spectrum of childhood cancer, many pathways converge on the same epigenetic regulators, such as PRC2. Understanding such convergent signatures and mastering regulators across subgroups or classes of cancers can change our approach to therapy. In addition to targeted therapeutics aimed at the epigenome, there is interest in understanding the epigenetic effects of traditional chemotherapeutic drugs. A key illustration of this comes from the recent observation that anthracyclines can induce histone eviction in cancer cells, independent of their ability to induce DNA breaks [239].

**Figure 7:**
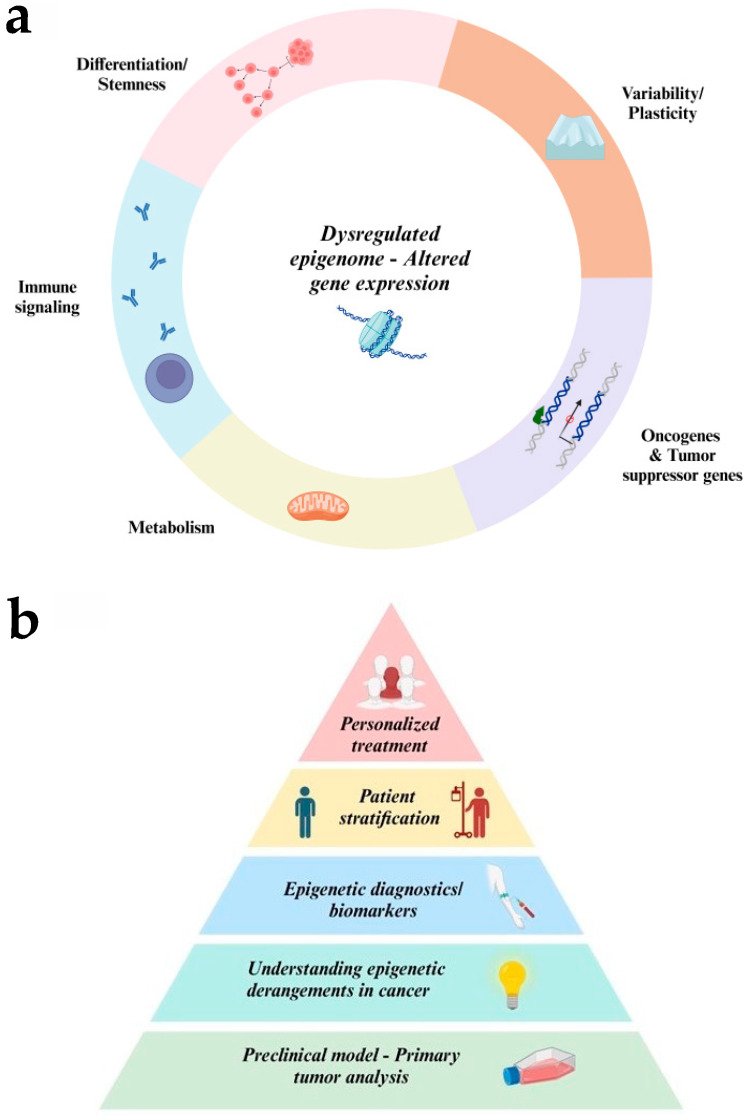
Principles of epigenetics in childhood cancer. (**a**) Epigenetic dysregulation impacts multiple pathways in pediatric cancer, including self-renewal and cell fate transitions, immune signaling, metabolic regulation, and epigenetic plasticity and variability. (**b**) Future prospects of epigenetics in cancer management. The potential of epigenetics in childhood cancer is reflected in a structured progression, resembling a pyramid, with each level serving as a stepping stone to the next. At the foundation, establishing efficient preclinical models and securing access to patient-derived primary samples provides insight into the epigenetic underpinnings of pediatric cancer. Building on this knowledge, the next step involves enhancing diagnostic capabilities through the development of epigenetically informed biomarkers and refined risk stratification tools. Finally, at the top of this progression, the ultimate goal is to deliver personalized epigenetic therapies tailored to the unique profiles of individual patients.

Epigenetic dysregulation has multifaceted effects in cancer cells, and intersects with immune regulation, metabolism, differentiation state, variability, and plasticity, in addition to the well-understood effects on the silencing of tumor suppressor genes and activation of oncogenes (Figure 7a) [240]. Epigenetic drugs can serve as modulators of immunogenicity in tumors. The potential for neoantigen expression and the induction of immune signaling has opened new avenues for the application of targeted immunotherapies to childhood cancer, which have not yet been fully realized [241,242].

A newly appreciated layer of complexity is the role of epigenetic stochasticity or variability as a driver of tumor evolution. Epigenetic variability enables transcriptional and phenotypic plasticity, enabling cells to sample diverse states that promote survival or facilitate treatment resistance [3,196]. Epigenetic drugs may offer a pathway to limit epigenetic plasticity for therapeutic benefit, such as by constraining the plasticity of cancer cells, opposing the emergence of resistance, or inducing therapeutic differentiation.

As noted in this review, the preclinical success of epigenetic therapeutics against model systems has not been effectively translated to the clinic. This raises questions about the adequacy of current model systems, such as xenograft studies lacking the interaction of cancer with its microenvironment or the immune system, but also about the outputs used to assess the efficacy of epigenetic therapeutics and the use of biomarkers to select cases that are more likely to respond to epigenetic interventions.

By gaining a deeper understanding of the epigenetic mechanisms underlying cancer, we can identify abnormalities that can be integrated into cancer diagnostics and biomarker development, stratify patients based on aberrant epigenetic profiles, and ultimately provide more personalized therapeutic interventions for pediatric cancer patients (Figure 7b) [243].

## 7. Conclusions

In this review, we highlight the intricate role of epigenetic mechanisms in childhood cancer, emphasizing pharmacologic strategies to target key nodes in epigenetic regulation. We identify gaps in the application of epigenetic therapies to pediatric cancers, as compared to those in adults, and we explore emerging concepts relevant to the epigenetic landscape of childhood cancer, including the role of epigenetic variability in driving cancer cell plasticity. Additionally, we emphasize the potential of rational combinations of epigenetic drugs with other therapies, particularly immune-based therapies. By advancing the understanding of dynamic epigenetic regulation in childhood cancer, we are poised for progress toward effective epigenetic therapeutics.

## Data Availability

No new data were created or analyzed in this study.
